# Pragmatic randomized controlled trial of providing access to a brief personalized alcohol feedback intervention in university students

**DOI:** 10.1186/1940-0640-7-21

**Published:** 2012-10-10

**Authors:** John A Cunningham, Christian S Hendershot, Michelle Murphy, Clayton Neighbors

**Affiliations:** 1 Centre for Addiction and Mental Health, 33 Russell St, Toronto, Ontario, Canada; 2 University of Toronto, Toronto, Canada; 3 University of Houston, Houston, TX, USA

**Keywords:** Randomized controlled trial, Problem drinking, Alcohol abuse, College, University, Internet-based intervention, eHealth, Brief intervention

## Abstract

**Background:**

There is a growing body of evidence indicating that web-based personalized feedback interventions can reduce the amount of alcohol consumed in problem drinking college students. This study sought to evaluate whether providing voluntary access to such an intervention would have an impact on drinking.

**Methods:**

College students responded to an email inviting them to participate in a short drinking survey. Those meeting criteria for risky drinking (and agreeing to participate in a follow-up) were randomized to an intervention condition where they were offered to participate in a web-based personalized feedback intervention or to a control condition (intervention not offered). Participants were followed-up at six weeks.

**Results:**

A total of 425 participants were randomized to condition and 68% (n = 290) completed the six-week follow-up. No significant difference in drinking between conditions was observed.

**Conclusions:**

Web-based personalized feedback interventions that are offered to students on a voluntary basis may not have a measurable impact on problem drinking.

**Trial Registration:**

ClinicalTrials.gov: NCT01521078

## Background

Heavy drinking on campus is one of the more troubling concerns facing university health-care workers, police, and administrators. Numerous surveys have identified the prevalence of heavy drinking [1,2]. Other statistics summarize the associated negative consequences, which range in severity from derailed academic aspirations, to increases in rape and violence, and drinking related fatalities [2-4].

Considerable efforts have gone into finding solutions for this endemic problem. Campus-wide policies and educational campaigns have been tried with mixed success [5]. Others have modified the tools and treatments validated in health-care settings to apply them to the concerns of heavy drinking on campus. These brief interventions have taken many forms, from face-to-face [6], written self-help materials [7], and more recently, Internet-based interventions (IBIs) [8].

Several advantages of IBIs have been identified. Once developed, they are inexpensive, can be made widely available, are accessible 24 hours a day, 7 days a week, and can circumvent some of the barriers to seeking help associated with concerns about stigma and labeling [9]. Several trials have been conducted evaluating the efficacy of these IBIs and, while the methodological rigor of the research varies [10], there is a growing body of research demonstrating the efficacy of IBIs to reduce heavy drinking on university campuses and in the general population [8,11-17]. The current project adds to this literature by evaluating whether providing *access* to a personalized feedback intervention (in this case the Check Your Drinking University version; CYDU) may result in short-term reductions in drinking. This issue is important as use of these interventions is often not a mandatory requirement for university students so the issue is whether making materials of this type available leads to any reductions in risky drinking.

## Methods

A list of 10,000 randomly selected university student emails was obtained from the university registrar’s office. Invitations to participate in a brief survey about student drinking on campus were sent (“Hello, we are conducting a brief survey (less than 5 minutes) asking students about their drinking alcohol. Your email address was selected by chance. We are looking for social drinkers to participate in this brief study. Please click this link to fill out the brief survey). Students were offered a $5 gift certificate from a large online store to complete the survey and were told that some students would be asked if they were interested in filling out another survey in six weeks’ time. Potential participants clicking on a unique link in the email were taken to a web page that contained a consent form. Those agreeing to participate were asked their age, sex, and the three item AUDIT-C measure (frequency of drinking, usual quantity of drinking, frequency of 5+ drinking days) [18]. Participants with AUDIT-C scores of less than four (cut-off for risky drinking) were thanked for their participation but were not invited to take part in another survey in six weeks. Participants who scored four or more on the AUDIT-C were invited to complete another survey in six weeks. Participants were told that they would receive an additional $10 gift certificate upon completion of the follow-up survey. Participants were also told that some students would be provided access to some other information about drinking on campus but that we would not know if they would be one of those students. Students indicated consent to participate in the follow-up survey (and to potentially receive additional information about drinking on campus – in this case access to the CYDU) by clicking on the link to agree to participate in another survey.

**Figure 1:**
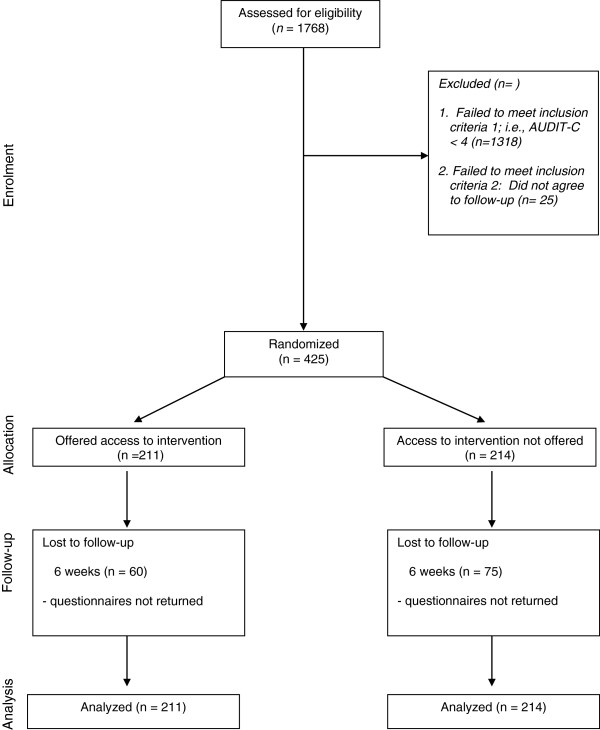
Consort diagram of the trial.

Eligible participants were then randomized to be provided access to the CYDU program or to not be provided access. Participants in the intervention condition (i.e., those provided access) were told, “we thought you might be interested in looking at a website that will let you see how your drinking compares with other university students. If you’re interested in seeing this website, please click on the link below.” Those who clicked on the link to go to the CYDU were then sent to the website http://www.CheckYourDrinkingU.net. This is an anonymous website so drinking data provided by these students on the CYDU was not recorded. Participants in the control condition (i.e., those not provided access to the CYDU) were thanked for their participation and told that they would be contacted again in six weeks’ time. At the six week time point, participants were contacted again by email inviting them to click on a unique link to fill out another survey about their drinking online (including a reminder that they would receive a $10 gift certificate for completing the survey). The follow-up survey asked the three AUDIT-C items phrased to refer to the last six weeks. The survey also asked if participants had completed the CYDU (included an option allowing them to state that they were not provided access). Participants were sent one reminder email if they did not respond within a week. The AUDIT-C was the only outcome variable. The outcome analysis was conducted using an Analysis of Covariance with baseline AUDIT-C scores used as the covariate. Missing data at follow-up were replaced by their respective baseline values. The study was approved by the standing ethics committee of the Centre for Addiction and Mental Health (see Figure 1).

### Check Your Drinking University version (CYDU)

The CYDU is a modified version of the Check Your Drinking screener (CYD) that has been made specifically relevant to university students. Both the CYDU and the CYD consists of a brief, anonymous, assessment (22 items) followed by a personalized feedback Final Report whose main components consist of normative feedback (comparing the participants’ drinking to others of a similar age, sex, and country of origin in the general population) and an assessment of the severity of the participants’ drinking concerns [19]. The CYD screener now has four randomized controlled trials demonstrating its efficacy in different settings [20-23], with problem drinkers displaying a six to seven drinks per week reduction (about a one third reduction from baseline) at three and six months after being provided access to the interventions [23]. Three of these trials have been with young adults, one in a workplace setting [20] and two with college students [21,22]. To-date, the CYDU has not been subjected to a randomized controlled trial although it has been available online for several years.

The CYD version was modified in several ways to create the CYDU. First, as relevance of comparison group seems to be an important component of the impact of normative feedback [24,25], we generated university student norms to use with the CYD-U. The USA norms come from the university student subsample of the National Household Survey on Drug Use and Health [26] and the Canadian norms from the 2004 Canadian Campus Survey [27]. Thus, students can receive feedback comparing their drinking to others of the same age (year by year), sex, and country of origin (US and Canada). Second, to increase the impact of information on the amount of alcohol consumed to the participant, graphical elements were added depicting the caloric content of the amount of alcohol consumed, the amount of weight gain and exercise required to off-set this weight gain, and alternative uses to put the amount of money spent on alcohol. The reader is invited to try the public access version of the CYDU at http://www.CheckYourDrinkingU.net.

## Results

Out of the 10,000 email invitations sent, 1768 potential participants clicked on the link and completed the baseline survey. A total of 450 participants scored four of more on the AUDIT-C and were invited to complete the six-week follow-up survey. Of these, 425 participants agreed and were randomized to experimental condition. Bivariate comparisons were made between participants in the intervention and control conditions and found no significant differences (*p* > .05). The mean (SD) age of the 425 participants was 22.6 (3.9), 52.5% were male, and the mean (SD) AUDIT-C score at baseline was 5.9 (1.8).

Of the 425 participants recruited at baseline, 68% (n = 290) provided six-week follow-up data. Loss to follow-up was not significantly different (*p* > .05) between experimental conditions, or by baseline age, sex, and AUDIT-C scores. An analysis of covariance was conducted, comparing AUDIT-C scores at six-week follow-up between intervention and control conditions with baseline AUDIT-C scores as the covariant. Missing data at follow-up were replaced by their respective baseline values. There was no significant differences between condition, F(1, 422) = 1.9, *p* = .17, Mean (SE) Intervention = 5.3 (0.1); Control = 5.4 (0.1). This analysis was rerun with only participants who provided follow-up data with similar results, F(1, 287) = 1.1, *p* = .30, Mean (SE) Intervention = 5.0 (0.1); Control = 5.2 (0.1).

### Use of the CYDU

At baseline, participants in the intervention condition were offered access to the CYDU website. Of the 211 participants in the intervention condition, 61% (128) said they were interested and were sent to the front page of the CYDU website. At follow-up, participants in the intervention condition were asked if they completed the CYDU and a total of 37 of the 151 participants followed-up (or 18% of the 211 eligible participants at baseline) said they had tried the CYDU screener.

## Discussion

Providing access to a personalized feedback intervention website does not appear to have an impact on college student drinking. The design of this study varied from other randomized controlled trials evaluating the efficacy of personalized feedback interventions because most other trials either automatically provided the feedback after participants recorded their baseline data or ensured that the participant received the feedback through some other means. In order to test the effectiveness of a web-based intervention when participation is voluntary, it is necessary to set up the evaluation trial so that the use of the intervention is voluntary (in this case by recruiting for a survey about drinking and offering a randomized half access to a website that would let them compare their drinking to other university students if they were interested). In addition, compensation for participating was minimal. When access is voluntary, only a minority of participants (less than 20%) who were offered access reported actually using the intervention, thus obviating the potential mean group effect for the intervention condition (i.e., those provided access to the intervention).

There were several limitations to this trial. The first is that there is no previous research evaluating whether the CYDU has an impact in a situation where all participants in the intervention condition actually receive the intervention. Thus, it is possible that the intervention itself is not effective. However, we judge this possibility to be unlikely as the CYDU is very similar to the general population version (the Check Your Drinking screener) which has been subjected to four randomized trials to-date including two with college students [20-23]. However, there is merit in conducting an efficacy trial with the CYDU in order to rule out this possibility. In addition, it is possible that the CYDU has an impact but that there is something about the way the CYDU was presented (or with the introductory page of the CYDU) that acted as a barrier to participants actually completing this web-based intervention. In the present study, 128 participants were redirected to the CYDU. However, only 37 participants actually reported completing the CYDU. Finally, some participants (27%, 115/425) were responding to the baseline survey using a mobile phone platform making it possible that they would be less likely to complete the CYDU which has been set up to be completed in a computer-based environment (thus contributing to the low completion rate).

The other primary limitation was that the study was underpowered. Based on one of the authors experience in other research trials (and from reports of the prevalence of problem drinking on university campuses), we estimated that sending email invitations to 10,000 college students would be sufficient to garner 2,000 participants completing the baseline survey. Of these, we estimated that 1,200 might be risky drinkers and that 1,000 would agree to participate in the trial. With a hoped for follow-up rate of 80%, we powered the study to detect a small difference on the AUDIT-C. In the current study, 10,000 email invitations were sent out, 1,768 responded and only 450 met criteria for risky drinking leading to a much lower than anticipated sample size. There are a number of possibilities for this low response rate. The first is that email invitations with links to websites might be met with growing suspicion given the possibility of computer viruses. Another possibility is that the incentive to participate was too small ($5 certificate). Still, the proportion of participants to the baseline survey was not that much lower than anticipated but rather the proportion of those who participated who were risky drinkers was overestimated. This may reflect the fact that the university campus under study had a substantial commuter population and comprised of a diverse population with a relatively low proportion of students who drank alcohol. Subsequent investigation and communication with campus administrators has confirmed that drinking rates on this campus are considerably lower than average. This limitation points to the need to evaluate web-based personalized feedback interventions in a variety of different college settings [28]. Irrespective of the smaller than anticipated sample size, the results were clear enough to establish that, if there was any difference between the groups at all, it was very minor and would not have reached statistical significance without a much larger sample size. A post hoc power calculation (80% power to detect a difference with an alpha of .05) revealed that roughly 2,400 participants per condition would be needed to detect the difference observed in this trial.

While no overall effects were observed in this trial, it is likely that CYDU or similar web-based feedback interventions might be most effective among specific subgroups of students. The present study included an extremely minimal assessment which precluded our ability to evaluate potential moderators. Future research might consider whether voluntary access to web-based feedback interventions might be most effective for students who view their drinking as problematic and/or who are considering changing their drinking.

Despite limitations, the negative results observed in this trial establishes the need to conduct more pragmatic trials of the potential real-world influence of web-based personalized feedback interventions before we can confidently make the claim that these interventions will have an impact on problem drinking in college students when these interventions are offered in a voluntary participation manner.

## Competing interest

The authors have no conflicts of interest to declare.

## Authors' contributions

JC, CH, MM, and CN contributed to the design of the study. JC conducted the analysis and wrote the draft of the manuscript. All authors read and approved the final manuscript.
